# An Innovative Method of Measuring Changes in Access to Healthful Foods in School Lunch Programs: Findings from a Pilot Evaluation

**DOI:** 10.1371/journal.pone.0146875

**Published:** 2016-01-22

**Authors:** Allison P. Hawkes, Stacy L. Weinberg, Ruth Janusz, Christine Demont-Heinrich, Richard L. Vogt

**Affiliations:** Tri-County Health Department, Greenwood Village, Colorado, United States of America; TNO, NETHERLANDS

## Abstract

**Introduction:**

A large local health department in Colorado partnered with 15 school districts to develop an approach to evaluate changes in access to healthy foods in reimbursable school lunches and a la carte offerings.

**Materials and Methods:**

School district nutrition managers were engaged at the start of this project. Health department dietitians developed criteria to classify food items as “Lower Fat and less added Sugar” (LFS) and “Higher Fat and more added Sugar” (HFS) based on the percentage of calories from fat and grams of added sugar. Lunch production sheets were obtained for two time periods, food items and the number of planned servings recorded. LFS and HFS planned servings were summed for each time period, and a LFS to HFS ratio calculated by dividing LFS planned servings by HFS planned servings. Additional analyses included calculating LFS: HFS ratios by school district, and for a la carte offerings.

**Results:**

In 2009, the LFS: HFS ratio was 2.08, in 2011, 3.71 (P<0.0001). The method also detected changes in ratios at the school district level. For a la carte items, in 2009 the ratio of LFS: HFS was 0.53, and in 2011, 0.61 (not statistically significant).

**Conclusions:**

This method detected an increase in the LFS: HFS ratio over time and demonstrated that the school districts improved access to healthful food/drink by changing the contents of reimbursable school lunches. The evaluation method discussed here can generate information that districts can use in helping sustain and expand their efforts to create healthier environments for children and adults. Although federal regulations now cover all food and beverages served during the school day, there are still opportunities to improve and measure changes in food served in other settings such as child care centers, youth correction facilities, or in schools not participating in the National School Lunch Program.

## Introduction

With rising rates of childhood obesity, there has been increasing national attention paid to the nutritional value of school lunches; from the USDA’s release of new school nutritional guidelines in 2012 [1] to Michele Obama’s focus on healthy eating for kids. Schools have risen to the challenge, offering more fresh fruits and vegetables, more whole grains and fewer processed foods.

Between March 2010 and June 2012, during an obesity-prevention-focused Communities Putting Prevention to Work (CPPW) grant, the health department partnered with the 15 school districts in its jurisdiction, serving more than 250,000 students. One of the goals of the grant was that by the grant’s end (in approximately two years), all 15 school districts in the department’s jurisdiction have enhanced, or adopted, and implemented new wellness and other policies that resulted in increased access to healthy food/drink, and/or limited availability of unhealthy food/drink.

To assist school districts, funding was provided to each school district for a school district grant coordinator whose primary responsibility was to enhance wellness policies and increase opportunities for physical activity and healthy eating. These coordinators worked with school district nutrition managers to increase access to healthy foods and decrease access to unhealthy foods in their school lunch programs; some had already begun these efforts, including one district that had started the process of moving toward increasing scratch-cooked items. The menu changes for each school district were determined by the school district’s nutrition manager and included (in part), increasing availability to and variety of fresh fruits and vegetables, switching to breads and rolls with a higher percentage of whole grains, and increasing the use of raw or minimally processed meat and potatoes in recipes (minimally processed food does not have a large amount of salt, sugar or fat added to it). The health department helped support those changes. For example, the health department identified grant funding for fresh fruits and vegetables and salad bars, and assisted with applications by providing letters of support. The department also encouraged managers to implement additional menu strategies. During this work, the school district partners looked to the health department to help them evaluate and measure the changes they were making to their school lunches.

To evaluate this effort, changes in school wellness policies and school lunches were assessed. The authors determined the assessment of school lunches and a la carte offerings required a method that 1) used existing records so that schools were not burdened, 2) quantified access to and assessed the change in food items in school lunches and a la carte offerings at two different time periods, 3) did not require special software for nutrient analysis because of the expense and not all school districts used it and 4) produced results that were easily understood by various audiences such as school board members, school leadership, or parents.

Previous researchers have examined school lunches using a variety of methods [2–4]. Bartholemew and Jowers [2] characterized entrees as low, moderate, or high fat, and compared an intervention school with a control school by measuring the percentage of children at each school who selected low, moderate, and high-fat entrees at lunch. Cullen and Watson [3] assessed the impact of a state nutrition policy by using food production records from 49 schools and calculating the average number of portions of food or beverage served per student per day, before and during the first year of implementation. Portions per student per day were analyzed for fruit, regular or non-fried vegetables, high-fat vegetables, and milk. A la carte items sold were also evaluated. Finally, Condon et al. [4] used data collected as part of a national study, and examined lunch menus from 397 schools (weighted to be nationally representative of public schools with National School Lunch Program) and tabulated the percentage of menus that offered specific food and food groups.

None of these methods met all the criteria we required, and thus one had to be developed. This paper describes the development of the analytic method, its application to school lunch food production records and a la carte menus, and demonstrates the usefulness of the analytic method in determining changes in access to healthy food/drink in school cafeteria lunches and a la carte offerings in the health department’s jurisdiction. Applicability to school districts nationwide and other organizations is discussed.

## Material and Methods

### School wellness policies

To evaluate changes to school district wellness policies, wellness policies and other pertinent district policies addressing student wellness were collected from each district for two time periods: prior to the grant in 2009, and at the end (March 2012). These policies will be collectively referred to as wellness policies throughout this manuscript.

For the 2009 policies, one person reviewed and assessed each school district’s wellness policies for references to 12 nutrition-related policy items selected from the state Wellness Policy Assessment Tool [5] that were most closely aligned with the goals of the grant. The health department added one other item to the review, which was “Guidelines have been established to encourage non-food or healthy food-related parties and celebrations in the classroom and teachers, staff, and families are notified annually.” For the 2011 policies, the same reviewer looked for references to those same 13 nutrition-related policy items that had either been added or strengthened since 2009.

### Developing and applying criteria to measure changes in access to healthy food/drink

To develop a process to measure changes in access to healthy food/drink, the 15 school district nutrition managers were engaged at the start of this project by meeting with them, obtaining their input on the process, and specifically, whether it would be feasible to use data they were already collecting on their daily food production records.

At the beginning of the grant in 2010 (school year 2009–2010), the nutrition standards in place for reimbursable school lunches were those that had been implemented in 1995 as part of the School Meal Initiative (SMI standards). The SMI standards were based on the 1995 Dietary Guidelines and required that **meals** provide no more than 30 percent of calories from fat and less than 10 percent of calories from saturated fat. The SMI also required that lunch provide one-third of the 1989 Recommended Dietary Allowances for energy (calories), protein, vitamins A and C, calcium and iron. Schools participating in the National School Lunch Program had five options available to them for meal planning. [6] The school districts in our jurisdiction used either food-based menu planning that required specific amounts of each food group be included in the daily school lunch [7] or nutrient standard menu planning which required USDA approved nutrient analysis software systems programmed to help plan menus that—when averaged over a school week—met the required level of calories, nutrients, and Dietary Guidelines for Americans for specific age groups. With nutrient-based menu planning, menu items are entered into the software and the only required food component is milk. [8]

The grant called for increasing access to “healthy” food and drink, and limiting availability of “unhealthy” food and drink. At the time, the USDA did not have a list of foods that met “healthy” criteria, so the health department conducted an extensive literature review that included among other sources, the Institute of Medicine’s Recommendations for Nutrient Targets and Meal Requirements for School Meals. [9] Dietary Guidelines for Americans, 2010, [10] and the USDA’s Proposed nutrition standards in the National School Lunch and School Breakfast programs 7 C.F.R. § 210 and 220. [11] The resulting criteria were aligned with national guidelines from the Institute of Medicine [9, 12] and the U.S. Departments of Agriculture (USDA) [10, 11, 13] and Health and Human Services [10] and on the evidence-based, GO-SLOW-WHOA criteria from CATCH^®^. [14]

The final criteria are defined in Table 1. For the purpose of measuring the relative changes in access to healthier food, two categories of food items were designated: one category (LFS) with a lower percentage of calories from fat (less than 40%) and/or a minimal amount of added sugar (less than 8 grams) and the other category (HFS) with a higher percentage of calories from fat (greater than or equal to 40%) and/or more added sugar (greater than or equal to 8 grams). The focus on fat and added sugar was determined after an initial review of several districts' menus, recipes, production menus and, if available, their nutrient analysis to determine the type of information available that could be utilized in a comparison. Since nutrient analysis was not required by USDA or the Colorado Department Education, many of the school districts did not have a nutrient analysis and used the USDA Meal Pattern for menu planning; so sodium content was not available. In order to determine an equitable measure to compare across all 15 districts, it was determined that the focus would be on fruit, vegetables and whole grains, which correlates to the fiber levels, and on the changes in types of individual food items offered that could be categorized by fat and added sugar. The comparison required a practical approach that could be applied to all 15 school districts with our very limited timeframe. In addition, focusing on fat and added sugar was consistent with the goal of the grant, which was obesity prevention. A cut-off of 40% of calories from fat rather than 35% was chosen because this method required examining individual food items, rather than an entire meal or a school day’s worth of food, both of which contain a variety of food items.

**Table 1:** Criteria developed by local health department dietitians for “Lower Fat” and “Higher Fat” food items in reimbursable school lunch meals.

Food Categories	Lower Fat/less added sugar (LFS)	Higher Fat/more added sugar (HFS)
**Entrees**	< 40% of calories from fat plus nuts, seeds, and nut/seed butters	≥ 40% of calories from fat, excluding nuts, seeds, and nut/seed butters
**Fruit**	< 8 grams added sugars/serving or in juice, light syrup, or no added sugar (includes fresh, canned and frozen)	≥ 8 grams added sugars/serving or in heavy syrup (includes fresh, canned and frozen)
**Vegetables** (includes potatoes and legumes)	< 15% of calories from fat (includes fresh, fresh cooked, canned and frozen vegetables)	≥ 15% of calories from fat (includes fresh, fresh cooked, canned and frozen vegetables)
**Grains**	< 20% of calories from fat; and < 8 grams added sugars/serving	≥ 20% of calories from fat; or ≥ 8 grams added sugars/serving

#### Applying the criteria to menu items on production sheets and a la carte items

Data were obtained on each district’s food preparation practices, recipes, and ingredients. School district nutrition managers were interviewed twice: once in the fall of 2010 (for the 2009–2010 school year) and again in the fall of 2011 (for the 2011–2012 school year). This information was then used by a single dietitian in coding the production records.

**Fig 1:**
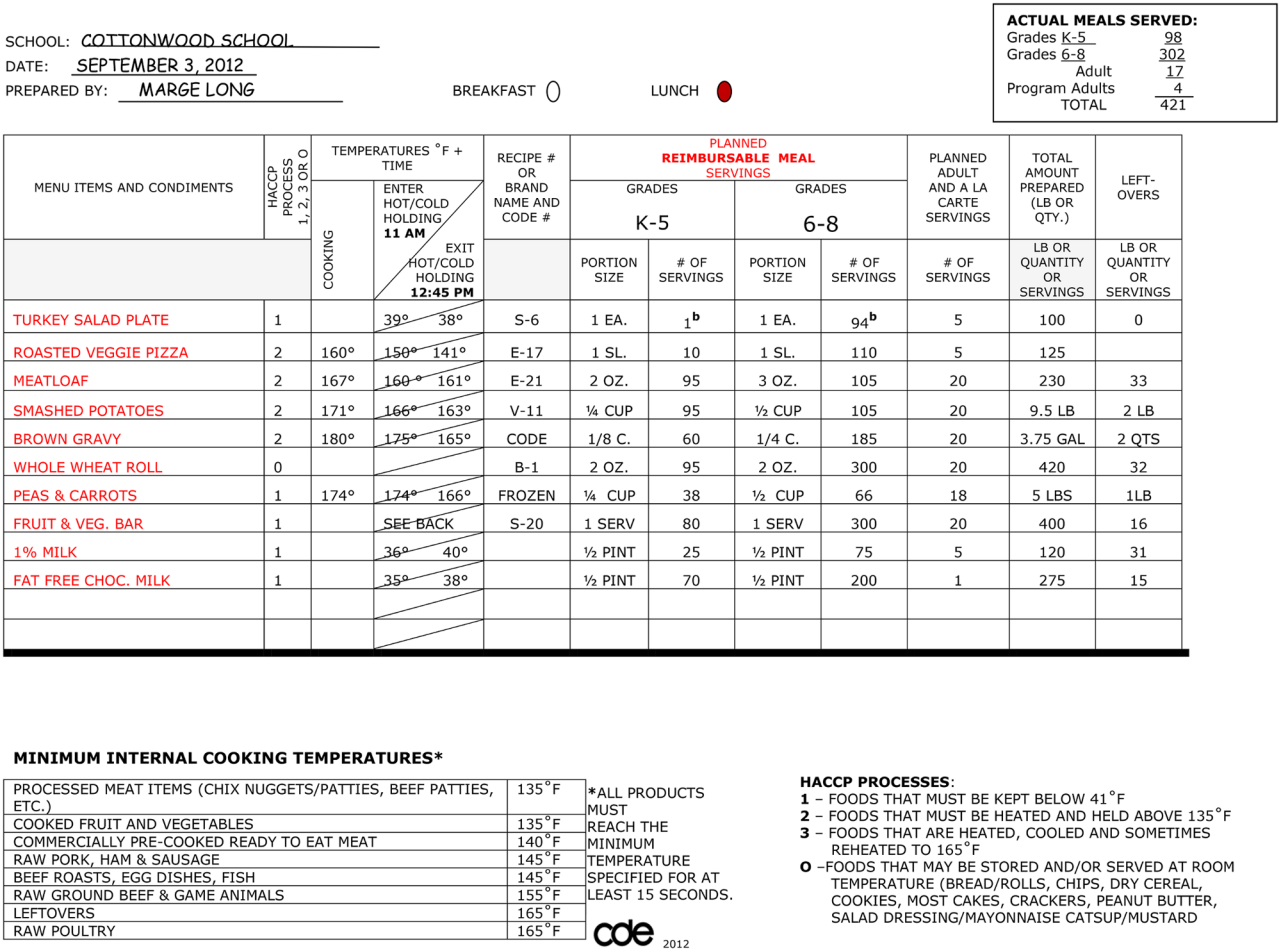
Daily production record. a. This production sheet is an older version from 2012 and may not include newer U.S. Department of Agriculture requirements. [13] b. The numbers were changed from their original values of 0 (grades K-5) and 95 (grades 6–8) for explanatory purposes (see text).

Production records are required documentation for school meals and include menu items offered, the number of servings prepared (also called planned servings), portion sizes, amount of food used, and the actual number of servings to students. Fig 1 depicts an example of a production record from 2012 [15], and was selected because it was similar to the ones reviewed for this evaluation. Production records, however, do vary from one district to the next, and may change over time to reflect new requirements from the USDA. Production records must be maintained for "reimbursable" meals for a period of 3 years. [16] Production records do not include all of the details, however, about how a menu item is prepared. Information from the district nutrition managers provided this level of detail. For example, in the health department’s jurisdiction, “frito pie” was an entrée that appeared on production sheets from several school districts in 2009 and 2011. For most districts, “frito pie” fell in the higher fat category. One district, however, went to scratch cooking in 2011 and created a healthier version of “frito pie”; the school district used low-fat cheese instead of a high-fat canned nacho cheese sauce, and increased the amounts of tomatoes and vegetables in the recipe.

Twenty consecutive days of lunch production records (one four-week cycle menu) were obtained from one randomly selected representative elementary and middle school in each school district for the time periods September–October 2009 and September–October 2011. The same schools were used during both time periods. A cycle menu is a series of menus planned for a particular period of time. The menu varies for each day of the cycle, and at the end of the cycle the menus are repeated.

One dietitian coded all of the production sheets. For a menu item to be included in the analysis the planned servings had to be equal to or greater than 10% of the number of students served that day. For example, if 98 students were served, and there was only one planned serving of a Turkey Salad Plate, then the Turkey Salad Plate was not included in the analysis (see Fig 1). Milk was excluded because districts had previously changed to fat-free or 1% low-fat milk.

For each menu item, the following information was abstracted from the production sheets and entered into a database: menu date, menu item description (e.g., hot dog, hamburger), food category (entrée, fruit, vegetable, and grain), the assessment code (LFS or HFS) and number of planned servings. If the reviewing dietitian needed additional nutrition information about a menu item in order to code it, she consulted the USDA nutrient database. [17]

In addition to the production sheets, the nutrition managers were requested to submit a list of a la carte items for each time period. Each item was assessed using the same criteria as for the reimbursable meals, and designated either LFS or HFS. The description of the item, the assessment code, and year of production sheet were entered into the computer.

All data were entered and analyzed in a Microsoft Access^®^ database. The accuracy of data entry was checked by printing a hard copy of the data entered into the computer and comparing it with the original hard copies of production sheets and a la carte lists. The consistency of coding items was checked using a query in Microsoft Access that compared the coding of menu items. Discrepancies were discussed with the coding dietitian.

#### Quantifying changes in access

The main outcome measure was quantifying changes in access to healthy food/drink for the entire jurisdiction using planned servings. Planned servings are the total number of servings that are prepared for reimbursable meals. Planned servings were chosen in contrast to actual servings (the number of portions served), because the actual servings are limited by the number of planned servings.

For each time period, the total number of LFS planned servings was determined by adding together the number of LFS planned servings for all of the food categories (entrée, fruit, vegetable, and grain); this process was then repeated for HFS planned servings. The number of LFS planned servings was then divided by the number of HFS planned servings to obtain a ratio. For each food category (entrée, fruit, vegetable, and grain) the ratio was obtained using the same method, but only included planned servings for the category of interest. A ratio greater than 1.0 indicates greater access to LFS menu items than HFS menu items, and a ratio less than 1.0 signifies the opposite. Any increase in the ratio is an improvement and means that access to “lower fat and less added sugar” food items has increased relative to food items with “higher fat and more added sugar,” but if the ratio is less than 1.0, then there is still greater access to HFS menu items overall.

A similar type of analysis was conducted for a la carte offerings; however, planned servings were not available for the vast majority of items on the a la carte menus. Instead, the total numbers of LFS a la carte items and HFS a la carte items was computed, and the ratio of LFS: HFS determined.

Secondary analyses included measuring changes in access to healthy food/drink by district and by school level (elementary schools vs. middle schools), and testing whether there was any correlation between the districts’ changes in access (as measured by changes in ratios) and the number of guidelines added or strengthened in the wellness policies.

Statistical significance of the differences in planned servings between the two time periods was assessed using a chi-square test. Correlations were tested using Spearman rank methods. A two-sided P value less than 0.05 was considered statistically significant. Statistical analyses were generated using JMP^®^ software, Version 11for Windows, copyright ^©^ 2013, SAS Institute, Inc. [18]

#### Demonstrating the method using example data

The method is demonstrated in the supporting information using an example with data from three days of production records from one school during two time periods.

## Results

### School wellness policies

During the grant, 14 of the 15 districts added at least one new nutrition-related policy item to their wellness policies (range 2–10 policy items). The one remaining district made a change pertaining to physical activity. In addition to adding new policy items, districts strengthened their existing nutrition-related policy items (range 0–7 policy items per district). By the end of the grant, there were a total of 64 new and 49 strengthened nutrition-related policy items in district wellness policies: by district, the total number of changed policy items ranged from zero to 12.

### School lunches

From 2009–2011, access to LFS food showed a statistically significant increase in reimbursable school lunches in the health department’s jurisdiction: the ratio of LFS: HFS menu items increased from 2.08 to 3.71 for all food categories combined, 0.79 to 1.23 for entrees, 27.94 to 309.94 for fruits, 2.07 to 5.40 for vegetables and 2.56 to 5.94 for grains which were all highly statistically significant (Table 2). Some of the changes in menu items that contributed to these results were a 50% reduction in the planned servings of French fries (51,000 to 23,000), hot dogs (11,000 to 6,000), and breaded chicken including nuggets, tenders, and patties (13,000 to 6,000). In addition, for a number of key LFS menu items, planned servings increased during that same time period; plain chicken increased from 4,500 planned servings to 11,000; plain beef, pork, or roast beef from 2,890 to 6,116, and fresh fruit from 50,324 to 81,575 planned servings.

**Table 2:** Change in planned servings of “Lower fat/less added sugar” and “Higher fat/more added sugar” school lunch menu items for all districts combined, by food group, 2009 compared to 2011.

	Planned Servings		Statistical Significance		Ratio^b^	
	2009	2011	*X*^*2*^	P	2009	2011
**All Food Groups Combined**						
LFS^a^	397685	466842	19,114 (*1 d*.*f*.*)*	<0.0001	2.08	3.71
HFS^a^	191565	125879				
**Results by Food Group**						
Entrees						
LFS	86211	110560	4,952 (*1 d*.*f*.*)*	<0.0001	0.79	1.23
HFS	109658	89747				
Fruits						
LFS	139677	157138	4,143 (*1 d*.*f*.*)*	<0.0001	27.94	309.94
HFS	4999	507				
Vegetables						
LFS	105513	124992	11,911 *(1 d*.*f*.*)*	<0.0001	2.07	5.40
HFS	51019	23132				
Grains						
LFS	66284	74152	4,950 *(1 d*.*f*.*)*	<0.0001	2.56	5.94
HFS	25889	12493				

^a^. LFS = “Lower fat/less added sugar”; HFS = "Higher fat/more added sugar”

^b^. Ratio = LFS planned servings/HFS planned servings

**Fig 2:**
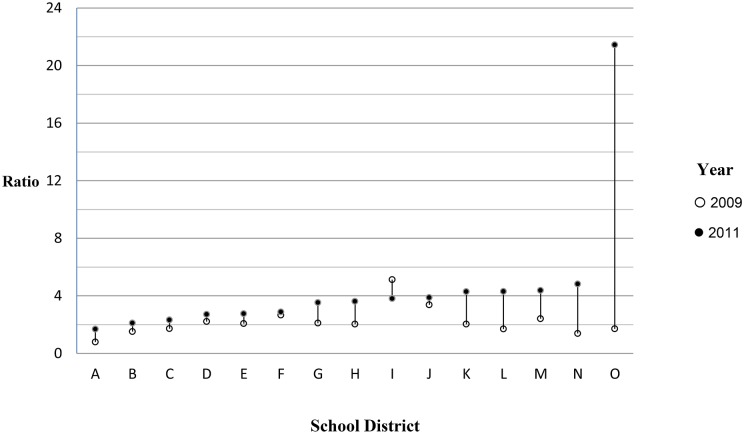
Change in LFS:HFS ratio of planned servings from 2009 to 2011, by school district. a. LFS:HFS ratio = Lower Fat/less added Sugar: Higher Fat/more added Sugar.

The method also detected a range of changes in ratios at the district level (Fig 2) ranging from an extremely positive change (District O) to a negative change (District I). The extremely positive change was observed in District O, which changed to scratch cooking; there were only two HFS food items served during the four-week cycle. French fries were served during one lunch in week two and cheeseburgers were served during one lunch in week four. The negative change observed for District I was the result of substituting HFS pizza (fat greater than or equal to 40% of calories) for LFS pizza (fat less than 40% of calories).

When the results were examined by school level, the findings were similar to those observed for the entire jurisdiction. For elementary schools, the ratio increased from 2.17 to 3.89 (*X*^*2*^ = 8067, 1 *d*.*f*., P<0.0001) and for middle schools, the ratio changed from 2.00 to 3.58 (*X*^2^ = 11073, 1 *d*.*f*., P<0.0001).

There was a statistically significant correlation between the magnitude of change in the LFS: HFS ratio and the number of nutrition policy items that the district strengthened or added. The correlation was assessed two ways: once by including the data from outlier District O (Spearman’s rho = 0.620, 13 d.*f*., P = 0.01) and once by excluding it (Spearman’s rho = 0.56, 12 *d*.*f*., P = 0.04).

### A la carte

Thirteen school districts responded to the request for their a la carte lists (86.7% response rate). Of the 13 districts, 11 districts (84.6%) offered a la carte. Of the districts with a la carte, all (100%) offered a la carte at the middle school and six (54.5%) offered it at the elementary school for a total of 17 schools. In 2009, 200 of a la carte items met the requirements for “LFS” and 377, “HFS” for a ratio of 0.53. The most commonly occurring food items that appeared on a la carte lists in 2009 were different types of entrées that could be purchased by themselves and not part of a meal (172 times). Various types of chips appeared 65 times, and cookies, brownies, etc., 50 times. In 2011, the ratio of LFS: HFS increased slightly to 0.61, but this difference was not statistically significant (*X*^2^ = 1.11, 1.*d*.*f*., P = 0.30).

## Discussion

The results showed that the school districts improved access to (availability of) healthy food/drink by changing the contents of reimbursable school lunches and adding or strengthening nutrition-related policy items to their districts’ wellness policies.

The health department developed partnerships with the leadership of the 15 school districts in our jurisdiction to increase access to healthy food/drink and create an innovative method to measure changes in access to healthier food items in school lunch programs. A method was developed that used existing records, so as not to create an undue burden on our school district nutrition manager partners, and did not require nutrient analysis software.

The reason for the changes is probably multifactorial. During the timeframe of the grant, new federal standards for reimbursable school lunches had been developed and were due to be implemented in the 2012–2013 school year. Thus it was not possible to determine whether the observed changes were due to the grant, and/or whether schools made changes in anticipation of the new federal requirements. While all school districts did receive financial support and exposure to various resources, the leadership involvement by school districts varied greatly. While it is difficult to objectively evaluate, it stands to reason that the districts with the most involved leaders tended to make the most number of changes in guidelines and food, while those with the least involved leadership, made fewer. Because of the study design, it is not possible to determine whether the change in wellness policies caused the change in ratio or the extent to which it contributed.

Existing tools found during the literature review focused on the number of food items on a school lunch menu [4] or the number of portions served. [3] However, for the purpose of measuring access, this evaluation found that examining just the number of “lower fat/less added sugar” and “higher fat/more added sugar” food items on the reimbursable school lunch menus could be misleading. For example, if a district adds two LFS items per meal, but plans on offering fewer servings of the LFS items and more servings of the HFS items, they haven’t increased access to healthier foods as much as it might seem on the face of it. In addition, planned servings were chosen in contrast to actual servings, because the actual servings are limited by the number of planned servings. The advantage of using the ratio of LFS: HFS is that it provides an easy way to interpret indicator of the relative access to healthier food. Increasing planned servings of healthier food is an important first step but, ideally, one would like the healthier choices to outnumber the less healthy choices. Additionally, the method does not depend on the number of students participating in school meals. For example, if 400 students are participating in school meals, and there are 300 planned servings of LFS entrées and 100 planned servings of HFS entrees, then the ratio of LFS: HFS is 3.0. If half of these students go on a field trip, presumably the nutrition service will only plan for 200 students. As long as the nutrition service plans to serve the same ratio of LFS: HFS entrees, then the ratio should not change. In this case, there would be 150 planned servings for LFS entrees, and 50 planned servings for HFS entrees for a ratio of 3.0. And the method is flexible; it can be applied to entire school lunch menus, or used in a more focused analysis of a specific food category (e.g., grains, fruits, vegetables) or a la carte. It does not require nutrient analysis software.

Since this evaluation was conducted, new standards have been implemented for reimbursable school lunches and are in the process of being implemented for competitive food and beverages sold outside the school meals program on the campus at any time during the school day. [19] Yet, there are still opportunities to improve and measure changes in access to healthy food/drink in other settings such as after-school functions, or concession stands. In addition, this method can be adapted to assess student acceptability of foods in schools where choices are offered; instead of using planned servings, one would use the actual number of servings. Other settings that use food production records—such as child care centers, youth correctional facilities, residential child care programs, schools for blind or deaf students, and schools or other residential institutions that do not participate in the National School Lunch Program—can also use this approach as described to measure changes in access to healthier foods in school lunch programs over time in order to demonstrate the results of the policy and systems changes being made by leadership. Other organizations such as hospitals can also use this approach to evaluate health promoting changes to their nutrition environments. For the evaluation of the grant, the criteria were based on the percentage of calories from fat (40%) and added sugar (8 grams). Others who adopt this method may wish to choose different criteria or modify the approach to include and compare other nutrients such as sodium (e.g., High sodium vs. Low sodium).

School leadership can use the information generated from this evaluation method to communicate changes in school food policies with students, parents, Boards of Education and other community partners about the significance of their efforts to create healthier food environments by making changes to school policies and food systems. In addition, they can use the information in grant proposals to potential funders to support continued work on healthier eating initiatives.

This method has limitations. This process does not measure the actual consumption of the school lunch offerings by students; it is strictly a measure of access to healthier offerings. However, measuring and quantifying improvements in access to healthier foods in school cafeterias will be instrumental in demonstrating intermediate outcomes along the path to the longer term outcome of reducing childhood obesity. Also, this method cannot be used in school districts in which families (or students) select the students’ lunch choices in advance, and the meals are then made and delivered.

In addition, applying this method to multiple school districts can be time-consuming. It is important to use data from an entire cycle of menus (regardless of the length of the cycle), because certain items may only be served once during the cycle, as was the case with the school district that went to scratch cooking.

## Conclusions

Increasingly, school districts are working to promote healthy eating and create environments that make healthy choices easier for students. As changes in obesity rates can take years to appear, it is critical that school officials be able to measure the changes in access to healthier foods as an intermediate measure of progress. The cost-effective evaluation method discussed here can generate information that districts can use in helping sustain and expand their efforts to create healthier school environments for children.

## Supporting Information

S1 Appendix (DOCX)

S1 Table Example of data from 3 days of production sheets from one school, and the coding applied by the evaluation Registered Dietician (R.D.), 2009 and 2011. (DOCX)
