# Supplementary material for: An Innovative Method of Measuring Changes in Access to Healthful Foods in School Lunch Programs: Findings from a Pilot Evaluation
Source: PLoS One. 2016 Jan 22;11(1):e0146875. doi: 10.1371/journal.pone.0146875 (PMC4723251; doi:10.1371/journal.pone.0146875)
Supplement: S1 Appendix — (DOCX) [file pone.0146875.s001.docx]

**S1. Appendix**

S1 Table shows an example of the data from three days of production sheets from one school, during two time periods. In 2009, LFS menu items appear 10 times on the production sheets and HFS menu items appear 10 times. Even though both appear 10 times on the menu, it is the number of planned servings that greatly affects the measurement of LFS (2350 planned servings) or HFS menu items (3450 planned servings) so that the resulting ratio is 0.68, signifying greater access to food items with a high percentage of calories from fat and/or more added sugar.

To determine the factors contributing to the overall ratio in 2009, the results can be examined by food category. Entrees and vegetables both have ratios less than one (0.23 and 0.18, respectively), showing many fewer planned servings of the LFS menu items than the HFS menu items in the entrée and vegetable food categories. Fruits and grains have ratios greater than 1.0 indicating greater access to LFS menu items than HFS menu items in the fruits and grains food categories.

In this example, the ratio of LFS to HFS menu items increases to 2.7 in 2011 for all food categories combined. Examining each food category separately, entrees increase to 1.94, fruit to 11.5, and vegetables and grains both increase to 1.83.
