# Supplementary material for: An Innovative Method of Measuring Changes in Access to Healthful Foods in School Lunch Programs: Findings from a Pilot Evaluation
Source: PLoS One. 2016 Jan 22;11(1):e0146875. doi: 10.1371/journal.pone.0146875 (PMC4723251; doi:10.1371/journal.pone.0146875)
Supplement: S1 Table — (DOCX) [file pone.0146875.s002.docx]

**S1 Table. Example of data from 3 days of production sheets from one school, and the coding applied by the evaluation Registered Dietician (R.D.), 2009 and 2011**

| **Data obtained from Production Sheet** | | |  | **Codes applied by evaluation R.D.** | |
| --- | --- | --- | --- | --- | --- |
| Date | Description of menu item | Planned Servings |  | Food Category^a^ | Rating^b,c,d^ |
| 9/1/09 | Chicken Nuggets | 200 |  | E | HFS |
| 9/1/09 | Pepperoni pizza | 350 |  | E | HFS |
| 9/1/09 | French fries | 550 |  | V | HFS |
| 9/1/09 | Dinner roll | 200 |  | G | LFS |
| 9/1/09 | Apples | 250 |  | F | LFS |
| 9/1/09 | Peach cobbler | 250 |  | F | HFS |
| 9/2/09 | Chicken Nuggets | 250 |  | E | HFS |
| 9/2/09 | Yogurt | 300 |  | E | LFS |
| 9/2/09 | Cinnamon roll | 300 |  | G | HFS |
| 9/2/09 | French fries | 550 |  | V | HFS |
| 9/2/09 | Carrot sticks | 150 |  | V | LFS |
| 9/2/09 | Grapes | 250 |  | F | LFS |
| 9/2/09 | Applesauce (no added sugar) | 250 |  | F | LFS |
| 9/3/09 | Hot Dog | 250 |  | E | HFS |
| 9/3/09 | Chicken Nuggets | 250 |  | E | HFS |
| 9/3/09 | Dinner Roll | 300 |  | G | LFS |
| 9/3/09 | Carrot sticks | 150 |  | V | LFS |
| 9/3/09 | French Fries | 500 |  | V | HFS |
| 9/3/09 | Oranges | 250 |  | F | LFS |
| 9/3/09 | Apples | 250 |  | F | LFS |
| 9/1/11 | Turkey sandwich | 300 |  | E | LFS |
| 9/1/11 | Grapes | 250 |  | F | LFS |
| 9/1/11 | French Fries | 300 |  | V | HFS |
| 9/2/11 | Chicken Nuggets | 250 |  | E | HFS |
| 9/2/11 | Spaghetti with marinara sauce | 300 |  | E | LFS |
| 9/2/11 | Carrot sticks | 150 |  | V | LFS |
| 9/2/11 | Clementines | 250 |  | F | LFS |
| 9/2/11 | Applesauce (no sugar added) | 250 |  | F | LFS |
| 9/2/11 | Cinnamon Roll | 300 |  | G | HFS |
| 9/3/11 | Chicken Nuggets | 225 |  | E | HFS |
| 9/3/11 | Tacos | 225 |  | E | LFS |
| 9/3/11 | Yogurt | 100 |  | E | LFS |
| 9/3/11 | Carrot sticks | 250 |  | V | LFS |
| 9/3/11 | Canned green beans | 150 |  | V | LFS |
| 9/3/11 | Apples | 400 |  | F | LFS |
| 9/3/11 | Peach cobbler | 100 |  | F | HFS |
| 9/3/11 | Dinner roll | 550 |  | G | LFS |

^a^ E=Entrée, F=Fruit, G=Grain, V=Vegetable;

^b^ LFS=Lower Fat/less added sugar, HFS=Higher Fat/more added sugar

^c^ 2009 Ratio for all food groups combined = 2,350 planned servings LFS/3,450 planned servings HFS = 0.68

^d^ 2011 Ratio for all food groups combined = 3,175 planned servings LFS/1,175 planned servings HFS = 2.7
